# *Cryptomeria japonica* var. *sinensis* Miquel (Chinese Cedar): A Comprehensive Review of Its Biology, Phytochemistry, Biotechnology, and Utilization

**DOI:** 10.3390/plants15162521

**Published:** 2026-08-20

**Authors:** Huwei Yuan, Liangye Huang, Junhan Guo, Tingting Jiao, Qinyuan Shen, Jiashuang Qiao, Jiaxin Hu, Yanyan Yin, Fuqiang Cui, Daoliang Yan, Yuanyuan Li, Jianfang Zuo, Kamran Shah, Bingsong Zheng

**Affiliations:** 1 State Key Laboratory for Development and Utilization of Forest Food Resources, Zhejiang A&F University, Hangzhou 311300, China; hwyuan@zafu.edu.cn (H.Y.); 2023602121036@stu.zafu.edu.cn (L.H.); guojh@stu.zafu.edu.cn (J.G.); 2025602122040@stu.zafu.edu.cn (T.J.); 2024202011016@stu.zafu.edu.cn (Q.S.); 2025602122079@stu.zafu.edu.cn (J.Q.); hujiaxin123@stu.zafu.edu.cn (J.H.); yinyan@stu.zafu.edu.cn (Y.Y.); fuqiang.cui@zafu.edu.cn (F.C.); liangsie@zafu.edu.cn (D.Y.); lyy@zafu.edu.cn (Y.L.); zuojianfang@zafu.edu.cn (J.Z.); 2 Zhejiang Key Laboratory of Non-Wood Forest Products Quality Regulation and Processing Utilization, Zhejiang A&F University, Hangzhou 311300, China

**Keywords:** *Cryptomeria japonica* var. *sinensis*, phytochemistry, molecular breeding, somatic embryogenesis, biomass utilization

## Abstract

*Cryptomeria japonica* var. *sinensis* Miquel (Chinese cedar) is an ecologically and economically vital evergreen conifer endemic to China. Renowned for its rapid growth and superior timber quality, the species has evolved from a traditional forestry resource into a multifaceted model species for phytochemistry and biotechnology. This review synthesizes recent advancements in its biology, chemical profiling, industrial utilization, and molecular breeding. Taxonomically and morphologically distinct from the Japanese variety, Chinese cedar exhibits robust physiological plasticity and complex transcriptomic responses to abiotic stresses. Phytochemically, the tree is a prolific reservoir of volatile essential oils, complex terpenoids, and flavonoids. These bioactive secondary metabolites demonstrate potent antimicrobial, antioxidant, and neuroprotective properties, driving broad applications in modern pharmacology and sustainable agrochemicals. Industrially, the timber is increasingly utilized in the fabrication of advanced engineered structural composites and circular bioenergy production. Concurrently, breakthrough biotechnological platforms including a chromosome-level reference genome assembly, multi-omics, somatic embryogenesis, and CRISPR/Cas9-mediated genome editing have advanced molecular breeding, accelerating the targeted development of climate-resilient and non-pollen-producing (hypoallergenic) elite cultivars. However, as global climate change and historical habitat fragmentation severely threaten ancient wild populations, this review advocates for integrated in situ and ex situ conservation strategies. By identifying critical research gaps in translational pharmacology, precision gene editing, and comparative genomics, this review provides a comprehensive foundation for understanding the biology, phytochemistry, biotechnology, and utilization of this invaluable botanical resource, offering a strategic roadmap for its sustainable management and commercial valorization.

## 1. Introduction, Taxonomy, and Botany

### 1.1. Overview

The family *Cupressaceae* is a widespread and ecologically important group of coniferous trees and shrubs. Within this diverse family, the genus *Cryptomeria* holds a unique evolutionary position. Fossil records indicate that *Cryptomeria* is an ancient Tertiary relict genus that was once widely distributed across Eurasia and North America during the Cenozoic era. Following dramatic climate fluctuations during the Quaternary period, its natural range contracted significantly. Today, it is recognized as a monotypic genus containing a single extant species, *Cryptomeria japonica* (L.f.) D. Don [1,2]. The word “cedar” itself carries a specific historical context. Early European settlers named the tree “cedar” because its appearance closely resembled that of the true Lebanese cedar (*Cedrus libani*). However, it is important to note that *Cryptomeria* belongs to the Cupressaceae family and is not closely related to true cedars (genus *Cedrus*). While the typical variety (*C. japonica* var. *japonica*) is famously known as the national tree of Japan, its mainland counterpart, *Cryptomeria japonica* var. *sinensis* Miquel (commonly known as Chinese cedar), represents a distinct and vital lineage endemic to southern China [2,3].

Chinese cedar is highly valued for its rapid growth, large stature, and high-quality wood. Because its timber is straight, durable, and naturally resistant to decay, the tree has been extensively cultivated for centuries. Today, it is one of the most important timber and afforestation species in the subtropical regions of China, playing a crucial role in modern commercial forestry, soil stabilization, and ecological restoration [4]. Beyond its forestry applications, *C. japonica* var. *sinensis* holds profound cultural and historical significance. Ancient trees, some estimated to be nearly a thousand years old, have been conserved by local communities in traditional “Fengshui forests” and around historic temples. These ancient populations serve as cultural symbols of longevity and act as important genetic reservoirs for conservationists [2,5].

In recent years, the species has garnered significant scientific attention for its rich phytochemistry and potential in biotechnology. The needles, twigs, bark, and sapwood of the Chinese cedar are abundant sources of bioactive secondary metabolites, particularly essential oils (EOs), terpenoids, and flavonoids [6,7]. These chemical compounds exhibit remarkable antioxidant, antimicrobial, and insecticidal properties. Consequently, extracts from *C. japonica* var. *sinensis* are highly sought after for applications in pharmacology, cosmetics, and green plant protection [6,8]. This diverse utility highlights the transition of Chinese cedar from a traditional timber resource to a valuable model species in plant biotechnology and chemistry. While recent, highly comprehensive reviews have addressed the biological activities and specialized metabolites of the broader *C. japonica* species [9], as well as the biotechnology and genetics of the Japanese cedar [1], a comprehensive synthesis focusing specifically on the Chinese lineage is lacking. This manuscript addresses this gap by focusing on the distinct biology, phytochemistry, and utilization of the mainland Chinese populations, while extrapolating foundational biotechnological and genomic advancements developed in the Japanese lineage that are applicable at the species level.

### 1.2. Literature Search Methodology

To conduct this comprehensive review, a systematic literature search was performed across major academic databases, including Web of Science, Scopus, PubMed, and Google Scholar, covering publications up to early 2025. The search strategy utilized combinations of Boolean keywords such as “*Cryptomeria japonica* var. *sinensis*” or “Chinese cedar” or “*Cryptomeria fortunei*” and “phytochemistry” or “secondary metabolites” or “somatic embryogenesis” or “molecular breeding” or “wood properties”. Selection criteria prioritized peer-reviewed research articles, authoritative taxonomic monographs, and comprehensive multi-omics reviews published primarily within the last 15 years to ensure the inclusion of up-to-date methodologies. Older foundational literature was included solely to establish historical taxonomy and validate ethnobotanical uses. Articles lacking rigorous peer review, grey literature, or studies presenting irreproducible extraction data were excluded from this review.

### 1.3. Taxonomy and Nomenclature

The taxonomic classification of the Chinese cedar has been a subject of long-standing botanical debate. Under the widely accepted international taxonomic framework, the genus *Cryptomeria* is classified as monotypic. Therefore, populations are divided geographically and morphologically into distinct varieties. The populations native to the Japanese archipelago are classified as the typical variety (*C. japonica* var. *japonica*). In contrast, the populations indigenous to mainland China were formally designated as a distinct botanical variety, *C. japonica* var. *sinensis*, by the Dutch botanist Friedrich Anton Wilhelm Miquel. This classification acknowledges the close evolutionary relationship between the mainland and island lineages while accounting for their regional adaptations [1,3].

The nomenclature of the Chinese cedar has been a subject of long-standing botanical debate. According to current taxonomic authorities, including Plants of the World Online (Kew), *Cryptomeria japonica* is recognized as the universally accepted species, with *C. japonica var. sinensis*, *C. japonica* subsp. *sinensis*, and *Cryptomeria fortunei* Hooibrenk treated as synonyms. However, because of its long-term geographical isolation from Japan and visually distinct morphological traits, many Chinese taxonomists historically argued that the mainland populations warranted distinct varietal or even species-level recognition [10]. As a result, the name *C. fortunei* or *var. sinensis* has been widely and persistently utilized in traditional Chinese forestry, agronomy, and phytochemical literature. Therefore, this review focuses specifically on the Chinese lineage and populations of *C. japonica*, while carefully cross-referencing literature to explicitly distinguish between the mainland lineage and the typical Japanese lineage [11].

**Figure 1:**
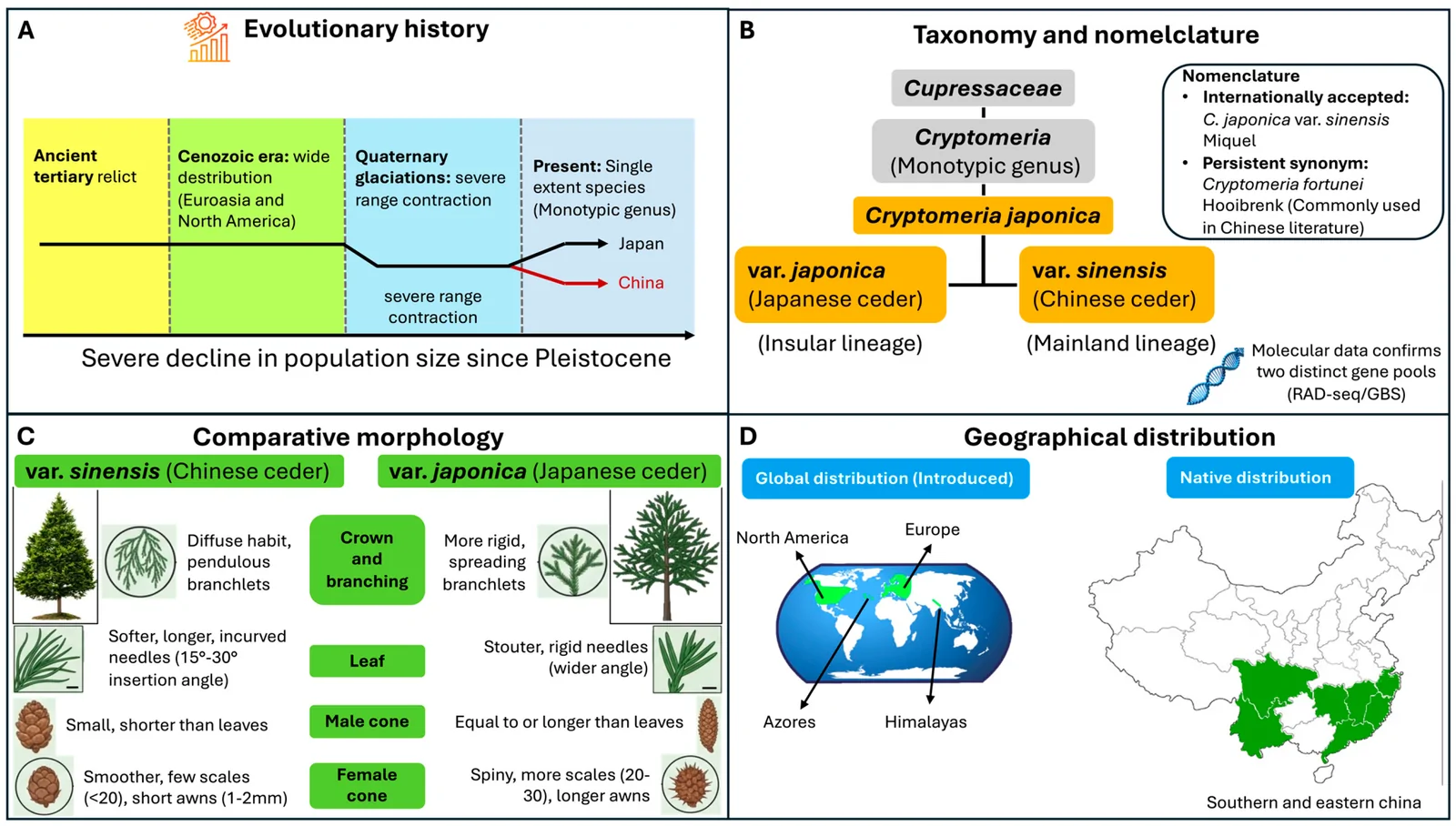
Evolutionary history, taxonomy, morphology, and distribution of *Cryptomeria japonica* var. *sinensis*. (**A**) Evolutionary history; (**B**) taxonomy and nomenclature; (**C**) comparative morphology; (**D**) geographical distribution.

Recent advancements in molecular phylogenetics and comparative genomics have successfully resolved this taxonomic ambiguity. Studies using advanced sequencing technologies, such as restriction site-associated DNA sequencing (RAD-seq) and genotyping-by-sequencing (GBS), have revealed strong genetic differentiation between the Chinese and Japanese populations [2,12]. These populations constitute two distinct gene pools that diverged due to ancient geographic isolation. Furthermore, complete chloroplast genome analyses show that *C. japonica* var. *sinensis* possesses a unique genomic architecture that lacks the typical inverted repeats (IR) found in many plants [11,13]. However, despite these clear evolutionary divergences, the overall genetic distance supports its classification at the varietal level. Thus, *C. japonica* var. *sinensis* remains the scientifically accepted botanical name [1,11] (Figure 1).

### 1.4. Morphology

**Figure 2:**
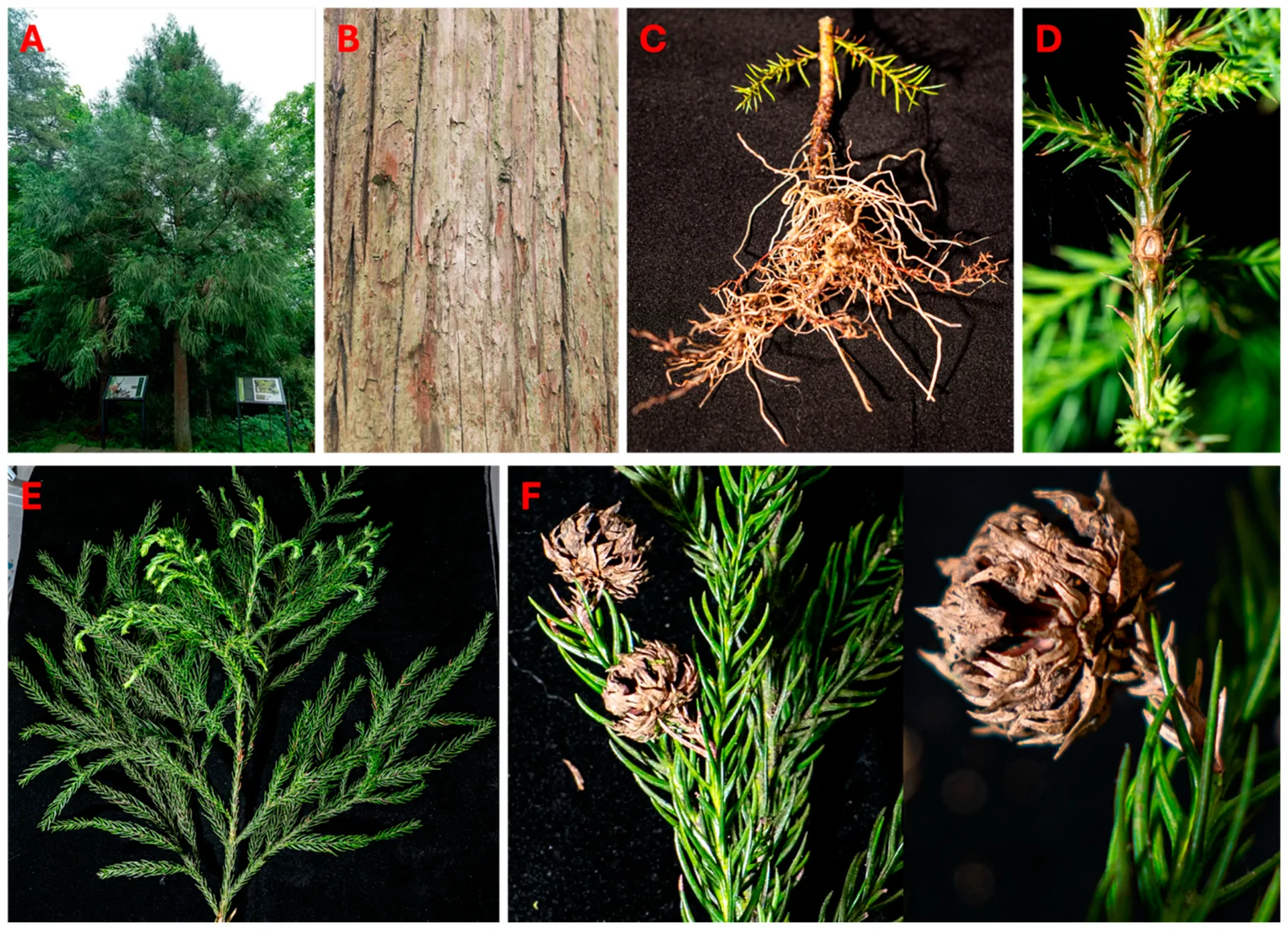
Phenotype of the Chinese cedar (*C. japonica* var. *sinensis*): (**A**) crown; (**B**) bark; (**C**) roots; (**D**) stem; (**E**) leaf branchlet; (**F**) seed cones.

*Cryptomeria japonica* var. *sinensis* is a massive, fast-growing evergreen conifer capable of reaching towering heights of up to 40 m. In its youth, the tree exhibits a strongly symmetrical and conical crown, which gradually expands into a broader, multi-domed canopy as it matures. The trunk is straight and covered in reddish-brown to dark gray fibrous bark that characteristically peels off in long, vertical strips [14] (Figure 2A,B,D). Below ground, the tree develops a highly adaptive fine root system (Figure 2C). Research indicates that the morphological traits of these fine roots, such as diameter and tissue structure, vary significantly based on their specific branch order. This structural plasticity allows the tree to optimize nutrient and water uptake in competitive subtropical soils [15].

Morphologically, the Chinese cedar exhibits several vegetative traits that easily distinguish it from the typical Japanese cedar. The overall habit of var. *sinensis* is more diffuse, characterized by slender, flexible, and distinctly pendulous (drooping) branchlets (Figure 2E). The foliar architecture also differs significantly. The awl-shaped, needle-like leaves of var. *sinensis* are notably softer, longer, and thinner than the rigid needles of var. *japonica* [14]. Furthermore, the leaves of the Chinese cedar are strongly incurved toward the stem and arise at much narrower branch insertion angles (typically 15 to 30 degrees), contrasting with the wider spreading needles of the Japanese variety [10,14]. Internally, these leaves contain specialized transfusion tissues that facilitate efficient water storage, a crucial anatomical adaptation for maintaining hydration at great canopy heights [16].

The reproductive structures of the tree also feature notable botanical variations. As a monoecious species, it bears separate male (pollen) and female (seed) cones on the same tree. The male cones of var. *sinensis* are small, scaly, and typically shorter than their subtending leaves. The female seed cones are woody, globular, and present distinct morphological deviations from var. *japonica* (Figure 2F). Specifically, the seed cones of Chinese cedar possess fewer cone scales, rarely exceeding 20 scales per cone, whereas the Japanese variety typically bears 20 to 30 scales [14]. Moreover, the distal projections (awns) on the bracts and cone scales of var. *sinensis* are notably shorter (usually 1 to 2 mm). This feature gives the mature Chinese cedar cones a much smoother and less spiny appearance [10,11] (Table 1).

### 1.5. Geographical Distribution

The native and historical habitat range of *Cryptomeria japonica* var. *sinensis* is primarily concentrated in the warm, humid subtropical regions of southern and eastern China. Natural occurrences and ancient relic forests have been documented across several provinces, including Fujian, Zhejiang, Jiangxi, Hunan, Sichuan, and Yunnan [3,12]. Within these native environments, the tree thrives in deep, well-drained, and acidic soils. It predominantly occupies subtropical and warm-temperate mountainous terrains and forested valleys. While it most frequently grows at elevations below 1000 m, it can successfully thrive in extended altitudinal ranges up to 2500 m under suitable microclimates. These regions provide the abundant rainfall, high humidity, and mild climatic conditions necessary for the optimal growth and natural regeneration of the species [2,5].

Historically, the natural distribution of Chinese cedar was much more expansive. Demographic history analyses suggest that the effective population size of the species has experienced a continuous and severe decline since the Pleistocene epoch, initially triggered by extreme climate fluctuations and glacial periods [2]. During the Holocene, this decline was further exacerbated by intense human activities, such as agricultural expansion and excessive logging for valuable timber. Consequently, primeval natural forests have been drastically reduced and fragmented. Today, native unmanaged stands are exceedingly rare, leaving only isolated ancient tree populations in remote, protected nature reserves, such as the Tianmushan National Nature Reserve in Zhejiang and Mount Lushan in Jiangxi [5].

Despite the reduction in its ancient wild populations, the regional and global distribution of *C. japonica* var. *sinensis* has been heavily expanded by artificial cultivation. It is now one of the most extensively planted coniferous species in southern China, forming the backbone of massive regional afforestation and commercial plantation programs [3,12]. Additionally, species distribution models under climate change scenarios predict that its highly adaptable nature will allow its suitable habitat to expand further toward the northeast and southwest in the coming decades [17]. Beyond its native borders, its robust adaptability and ornamental appeal have led to its successful introduction into various temperate and subtropical regions worldwide. It is now cultivated in parts of Europe, North America, the Azores, and the Himalayas, significantly broadening its global ecological footprint [6,14].

## 2. Biology: Ecology, Physiology, and Genetics

### 2.1. Ecological Requirements

**Figure 3:**
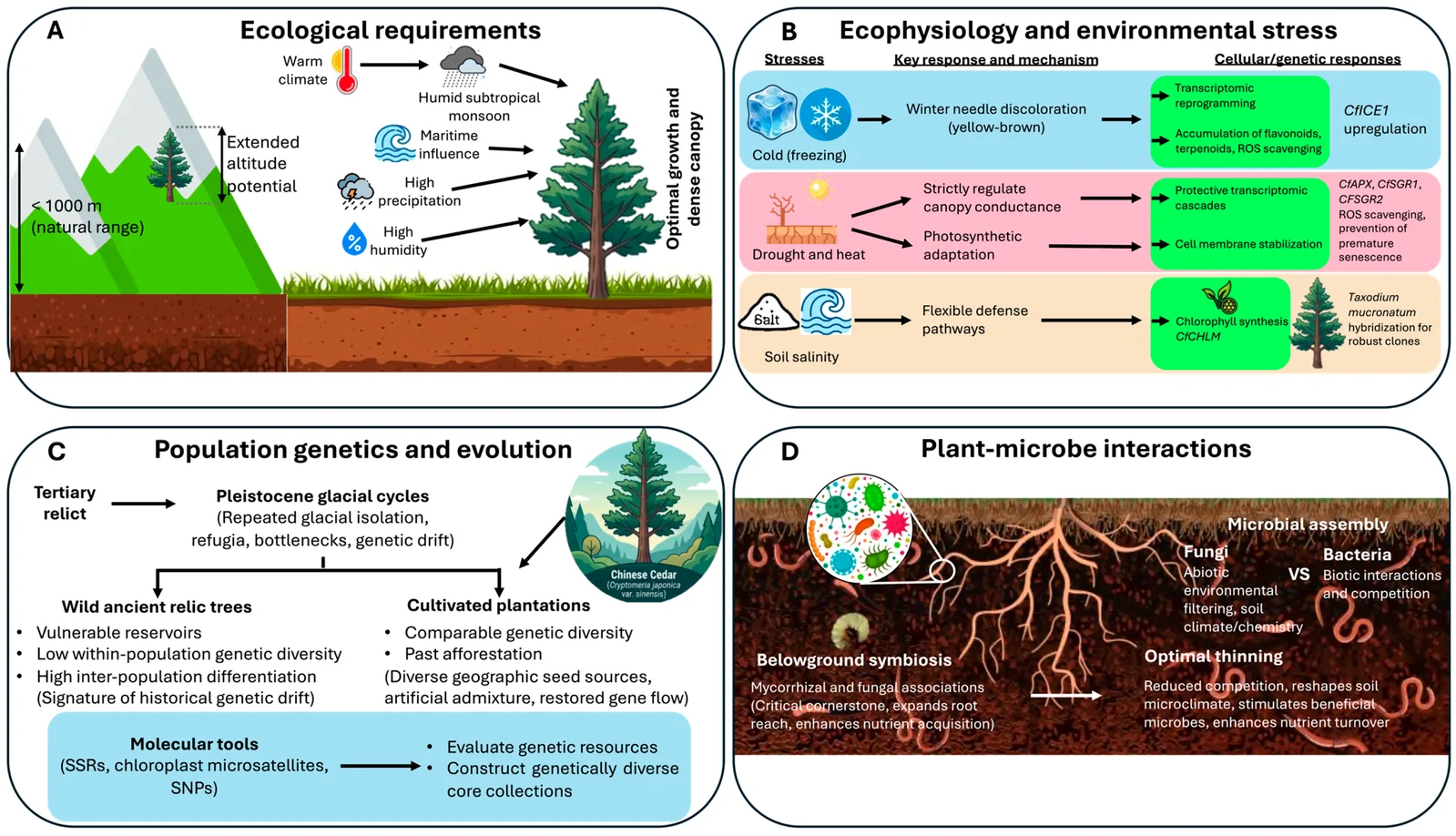
Ecological requirements, ecophysiological and environmental stress, population genetics, evolution and plant–microbe interactions of *Cryptomeria japonica* var. *sinensis*. (**A**) Ecological requirements; (**B**) ecophysiological and environmental stress; (**C**) population genetics and evolution; (**D**) plant–microbe interactions.

Within its native habitats, Chinese cedar plays a critical role in regional afforestation, ecological restoration, and timber production [18,19] (Figure 3). The specific altitudinal gradients are known to directly influence its climate-growth sensitivity and species-specific adaptation strategies [19].

The species performs optimally in a warm, maritime-influenced environment. Because of its moisture-dependent nature, it requires abundant precipitation and high ambient humidity to sustain its rapid growth and dense canopy. Its sap flow velocity and transpiration responses are highly sensitive to meteorological factors, demonstrating pronounced spatial and temporal variations based on environmental moisture [20,21]. During periods of intense summer heat and reduced precipitation, the species can even exhibit a bimodal pattern of intra-annual wood formation, as cambial activity temporarily pauses to conserve water resources [22].

Regarding soil preferences, *C. japonica* var. *sinensis* generally thrives in deep, well-drained, and nutrient-rich soils. However, the species exhibits a robust capacity for topographic adaptability, allowing it to be cultivated in challenging and degraded landscapes. Chinese cedar has been successfully utilized in the karst returning farmland-to-forest regions of southwest China. In these environments, trees dynamically adjust their stem sap flow, the core process of plant water transport, to cope with the unique hydrogeological constraints [23]. Despite this adaptability, managing proper soil properties through silvicultural practices, such as controlled stand thinning, remains essential to maintaining optimal long-term forest productivity [24].

### 2.2. Ecophysiology and Environmental Stress

Chinese cedar frequently faces severe abiotic stresses, and its physiological and cellular adaptation mechanisms have been extensively documented. Despite its preference for warm climates, winter cold and freezing temperatures present persistent threats. A hallmark phenotypic response to cold stress is winter needle discoloration, where its normally evergreen foliage turns yellowish-brown. This shift represents an active cold acclimation strategy driven by profound transcriptomic and metabolomic reprogramming, resulting in a massive accumulation of flavonoids, terpenoids, and photoprotective pigments that scavenge reactive oxygen species (ROS) [25,26]. Furthermore, key regulatory genes such as the inducer of CBF expression 1 (*CfICE1*) are significantly upregulated, conferring enhanced cellular resistance to chilling injury [27].

Drought and extreme heat pose combined threats to the physiological homeostasis of the species. Under prolonged drought or water deficit conditions, Chinese cedar curtails water loss by strictly regulating canopy conductance and adjusting photosynthetic traits to prevent severe photoinhibition [28,29]. During extreme summer heat, the species initiates protective transcriptomic cascades that stabilize cell membranes and maintain physiological integrity [30]. To mitigate heat-induced oxidative damage, genes such as the cytosolic ascorbate peroxidase gene (*CfAPX*) rapidly scavenge excess ROS, enabling broad-spectrum tolerance to thermal and osmotic stresses [31]. Additionally, stay-green genes (*CfSGR1* and *CfSGR2*) mediate hormone signaling and abiotic stress responses to prevent premature leaf senescence [32].

Soil salinity is an emerging environmental challenge, particularly in degraded coastal or intensively managed plantation sites. While Chinese cedar is not naturally halophytic, its innate genetic architecture provides flexible defense pathways. Research demonstrates that the overexpression of native genes like *CfICE1* and *CfCHLM,* which promote essential chlorophyll synthesis, significantly bolsters salt stress tolerance in transgenic models by sustaining physiological efficiency and osmotic balance [33,34]. Furthermore, hybridization efforts with highly salt-tolerant species like *Taxodium mucronatum* have successfully generated robust hybrid clones capable of withstanding extreme soil salinity while maintaining stable growth and physiological functioning [35].

### 2.3. Population Genetics and Evolution

As a tertiary relict plant in East Asia, the phylogeography and demographic history of *Cryptomeria* were profoundly shaped by historical climatic oscillations. Population genomic studies utilizing RAD-seq reveal that repeated Pleistocene glacial cycles forced the species into isolated refugia, causing significant demographic bottlenecks and genetic drift [36,37]. Consequently, *C. japonica* var. *sinensis* exhibits strong geographical variation and pronounced genetic differentiation among its natural populations [3]. This historical fragmentation, compounded by geographic barriers and restricted natural gene flow, has established unique genetic structures across its native distribution in China. A comprehensive species-wide review by [38] directly compared Japanese and Chinese populations, providing a vital framework for understanding this divergence. The study highlighted how historical disturbances, particularly the Last Glacial Period, shaped the genetic structure and local adaptation of the species, confirming a clear genetic differentiation between the insular Japanese and mainland Chinese lineages [38].

A central focus of population genetics in Chinese cedar involves comparing wild ancient relic trees with widespread commercial plantations. Ancient tree populations serve as essential, yet highly vulnerable, reservoirs of the species’ ancestral genetic signatures. However, due to historic anthropogenic pressures and prolonged isolation in refugial habitats, these ancient stands display alarmingly low within-population genetic diversity and high inter-population genetic differentiation [2,39]. This phenomenon reflects a classic signature of historical genetic drift, raising concerns about the localized extinction of unique adaptive alleles in these fragmented relic populations.

Paradoxically, modern, large-scale cultivated plantations harbor levels of genetic diversity that are comparable to those of the remaining ancient wild stands. This unexpected resilience is largely attributed to past afforestation programs that sourced seeds and propagation materials from diverse geographic origins across eastern and southern China [40]. This artificial admixture has inadvertently restored gene flow and preserved standing genetic variation within cultivated environments. To facilitate modern breeding and conservation, researchers have developed advanced molecular tools, including simple sequence repeats (SSRs), chloroplast microsatellites, and single nucleotide polymorphisms (SNPs), to evaluate genetic resources and construct genetically diverse core collections [10,12,41,42].

### 2.4. Plant–Microbe Interactions

The growth, nutrient acquisition, and environmental resilience of Chinese cedar are inextricably linked to its complex interactions with the soil microbiome. The root system architecture provides a dynamic ecological niche where physical structures and developmental processes actively shape the local microbial community [43]. Extensive regional surveys across East Asia demonstrate that fungal and bacterial communities in the *Cryptomeria* rhizosphere assemble via fundamentally different ecological processes. Fungal community composition is predominantly driven by abiotic environmental filtering, such as local variations in soil climate and chemistry, whereas bacterial communities are more heavily influenced by biotic interactions and localized competition [44].

Symbiotic mycorrhizal and fungal associations are a critical cornerstone of Chinese cedar’s belowground ecology. Because the species is frequently cultivated in competitive environments, it relies on complex microbial and rhizosphere symbioses to expand the absorptive reach of its root system and aid in nutrient uptake. These vital fungal networks effectively colonize the root zone, enhancing the acquisition of essential mineral nutrients. Cultivation strategies that amend the soil, such as the application of controlled-release fertilizers or glutathione, can further influence seedling growth and understory dynamics, highlighting the delicate balance between nutrient availability, root symbioses, and competing flora in the rhizosphere [45,46].

Forest management practices exert a cascading influence on these vital plant–microbe interactions and the overall health of the soil microbiome. Silvicultural interventions, such as controlled stand thinning, directly alter the soil microclimate and modify understory vegetation diversity. These ecological changes subsequently reshape the soil’s physicochemical properties and the structural composition of the microbial community. Studies investigating different thinning intensities in *C. japonica* var. *sinensis* plantations have shown that optimal thinning modifies the rhizosphere environment, stimulates beneficial microbial populations, and ultimately enhances soil nutrient turnover, bolstering the long-term resilience and productivity of the plantations [24].

## 3. Phytochemistry and Secondary Metabolites

### 3.1. Chemical Profiling

The Chinese cedar serves as a rich biological reservoir of diverse secondary metabolites [47,48]. The primary classes of phytochemicals identified within this species encompass a broad spectrum of volatile organic compounds (VOCs) (mainly monoterpenes and sesquiterpenes) and heavy non-volatile constituents, such as complex terpenoids (diterpenes and sesquiterpenes), flavonoids, and lignans [48,49]. These naturally occurring compounds play essential ecological roles, providing robust defense mechanisms against environmental stressors, pathogens, and herbivores, while concurrently offering substantial commercial value in the pharmaceutical, cosmetic, and timber industries [48]. Modern phytochemical profiling has demonstrated that the plant’s secondary metabolism is highly complex, yielding bioactive molecules that vary significantly across its anatomical structures (Table 2).

The distribution of these phytochemicals is highly tissue-specific, fluctuating considerably among the needles, bark, seed cones, and woody parts of the tree [49,50]. The needles (foliage) and seed cones serve as concentrated sources of EOs and protective flavonoids, which are crucial for the plant’s active interaction with its environment [49,58]. In contrast, the bark is a well-documented source of heavy, resinous, non-volatile terpenoids, notably yielding a rich profile of abietane-type diterpenes [52]. Concurrently, the woody tissues comprising the sapwood and heartwood accumulate distinct volatile oils alongside unique phenolic polymers known as norlignans [50,57]. These non-volatile components actively contribute to the timber’s characteristic dark color and its renowned natural resistance to biological decay.

To systematically isolate and profile these distinct phytochemical classes, researchers employ a combination of traditional and modern extraction techniques tailored to the target compounds [49]. EOs are typically obtained using hydrodistillation or water-steam distillation, with recent methodologies utilizing sequential fractionation to selectively separate aromatic components based on their volatility [59]. For the extraction of heavier, non-volatile secondary metabolites, organic solvent extraction utilizing methanol, ethanol, or acetone remains the standard approach [60]. Following extraction, these crude fractions are subjected to advanced analytical platforms to ensure precise structural elucidation and comprehensive chemical profiling. The identification of these compounds relies on highly specific analytical methodologies. Volatile components (EOs) are primarily analyzed using Gas Chromatography-Mass Spectrometry (GC-MS), where compound identification is achieved by comparing retention indices and mass fragmentation spectra against established databases (e.g., NIST libraries). For non-volatile, high-molecular-weight metabolites such as flavonoids and lignans, Liquid Chromatography-Mass Spectrometry (LC-MS) is favored to determine precise molecular weights and fragmentation patterns. Furthermore, for the absolute structural elucidation of novel or highly complex molecules such as the newly discovered dimeric and skeletally rearranged diterpenoids from the bark, researchers rely heavily on 1D and 2D Nuclear Magnetic Resonance (NMR) spectroscopy (including HSQC and HMBC techniques) to map exact atomic connectivity and stereochemistry [49,60].

### 3.2. Volatile Organic Compounds (VOCs)

VOCs constitute a major and highly valued fraction of the secondary metabolites in Chinese cedar, predominantly extracted in the form of EOs [49,59]. The overall yield and physical properties of these EOs depend heavily on the specific anatomical part being processed and the chosen extraction parameters. Hydrodistillation is the most widely adopted extraction technique, commonly yielding an aromatic, pale-yellowish oil from fresh foliage [49]. Recent optimizations in distillation timeframes have demonstrated that modifying the extraction duration facilitates the sequential separation of distinct EO fractions [59]. For example, shorter distillation times typically release lighter monoterpenes, while extended extraction periods are required to efficiently isolate heavier, oxygenated sesquiterpenes without causing thermal degradation to the bioactive constituents [49,59].

The chemical composition of Chinese cedar EOs is inherently complex, dominated predominantly by monoterpenes and sesquiterpenes [48,50] (Table 3). While the precise chemical makeup changes depending on the tissue analyzed, signature compounds such as ⍺-pinene, cedrol, and elemol are consistently identified as major constituents across various studies [50]. The highly volatile monoterpene ⍺-pinene is primarily responsible for the characteristic fresh, pine-like aroma of the foliage. Conversely, cedrol and elemol are heavier, oxygen-containing sesquiterpenes found abundantly in both the leaves and the woody tissues [48,49]. These specific compounds are highly sought after by the fragrance and pharmaceutical industries due to their distinct olfactory profiles and well-documented biological activities, including broad-spectrum antimicrobial and anti-inflammatory properties [51] (Table 3).

The VOC profile exhibits remarkable variation across different plant organs, reflecting the diverse physiological functions of these emissions [49]. EOs derived from the fresh needles are typically characterized by high concentrations of ⍺-pinene, sabinene, and elemol [49,50]. In stark contrast, EOs extracted from the sapwood and heartwood are notably rich in heavier sesquiterpenes and specific diterpenes, such as sclarene and cupressene, which contribute to the timber’s natural durability [50]. Furthermore, the reproductive organs, including female cones, produce unique volatile profiles containing specialized oxygenated hydrocarbons not commonly found in the vegetative tissues [58]. This organ-specific partitioning of VOCs underscores the sophisticated metabolic compartmentalization within the tree.

### 3.3. Non-Volatile Compounds

Beyond its aromatic volatile emissions, *Cryptomeria japonica* var. *sinensis* is a prolific producer of diverse non-volatile secondary metabolites, with terpenoids representing the most extensively studied structural class [48]. Phytochemical investigations targeting the tree’s bark have been particularly fruitful, leading to the successful isolation and structural elucidation of numerous complex non-volatile metabolites. While traditional research primarily identified conventional diterpenes and sesquiterpenes, recent advancements utilizing high-resolution mass spectrometry and multi-dimensional nuclear magnetic resonance (NMR) spectroscopy have dramatically expanded the known chemical space of this species. Researchers have identified a vast array of diterpenoids, including classic abietane-type diterpenes [52,61,62], pimarane [54], isopimaric acid derivatives [63], and phyllocladane-type diterpenoids [64]. The abietane skeleton is frequently further functionalized, yielding highly oxidized derivatives such as 7-oxoabietane-type diterpenoids [65] and skeletally rearranged *seco*-abietanoids [66,67]. Furthermore, the bark is an exceptional source of higher-order and complex dimeric structures, yielding novel dimeric abietane-type diterpenoids [68], bioactive dimeric abietanoid peroxides [60,69], and rare sesquarterpenoids [70]. The biosynthesis of these complex terpenoids relies on the coordinated action of specific terpene synthases and cytochrome P450 enzymes.

Biologically, many of these newly isolated bark extractives exhibit remarkable pharmacological and agrochemical properties. Ecologically, abietane and pimarane diterpenoids demonstrate potent antifungal activities against destructive wood-decay fungi, highlighting their integral role in the timber’s natural defense and their potential as eco-friendly wood preservatives [52,54]. Furthermore, these bark diterpenoids possess notable enzyme-inhibitory capacities with significant therapeutic potential. Specific *seco*-abietanoids, 7-oxoabietanes, and isopimaric acid derivatives have shown prominent inhibitory activity toward xanthine oxidase, a key enzyme implicated in hyperuricemia and gout [63,65,66]. Certain dimeric abietanoid peroxides exhibit dual inhibitory activities, effectively targeting both xanthine oxidase and angiotensin-converting enzyme (ACE) [69], while other dimeric analogs and complex diterpenoids display broader enzymatic inhibition alongside previously documented cytotoxic properties [48,60,68].

Flavonoids represent another crucial class of non-volatile metabolites, synthesized predominantly within the foliage of the tree via the well-conserved phenylpropanoid pathway [47,56]. Recent multi-omics studies, integrating both transcriptomics and metabolomics, have meticulously mapped the complex biosynthetic networks responsible for flavonoid production in this species [4,47]. These pathways are tightly regulated by specific transcription factors, such as the R2R3-MYB and NAC families, which dictate the synthesis and cellular accumulation of complex phenolic compounds [4]. Consequently, diverse flavonoids including catechin, epicatechin, quercetin, and various flavonoid glycosides have been isolated from the needles, where they serve as vital antioxidants that neutralize ROS and bolster the plant’s physiological resilience [47,56].

In the internal woody tissues, particularly within the heartwood, the non-volatile extractives are distinctly dominated by a specialized group of lignans known as norlignans [57]. Norlignans possess a unique C6-C5-C6 diphenylpentane carbon skeleton and are synthesized during the natural process of heartwood formation as ray parenchyma cells undergo programmed cell death [57]. Agatharesinol is one of the most prominent norlignans isolated from *Cryptomeria* species. The localized biosynthesis and subsequent polymerization of these norlignans not only impart the characteristic reddish-dark coloration to the cedar’s core but also significantly enhance the structural integrity and natural resistance of the timber against biological degradation, distinguishing it from the surrounding sapwood [57].

### 3.4. Seasonal and Spatial Variations

The biosynthesis and accumulation of secondary metabolites in Chinese cedar are highly dynamic processes that fluctuate significantly in response to seasonal environmental changes [56,71]. During the cold winter months, the needles frequently undergo a visible phenotypic discoloration, shifting from dark green to yellowish-brown [71]. Integrated transcriptomic and metabolomic analyses have revealed that this discoloration is directly linked to internal metabolic reprogramming. Cold acclimation drastically upregulates the expression of key genes within the flavonoid and terpenoid biosynthesis pathways, leading to a massive accumulation of protective flavonoids within the overwintering tissues [47,56]. This seasonal phytochemical surge serves as a crucial adaptive mechanism, mitigating cold-induced oxidative damage and significantly enhancing the tree’s overall freezing tolerance [47].

Geographical origin and local spatial factors also exert a profound influence on the chemical profiling of the tree, giving rise to distinct chemotypes [6,72]. Due to differences in local climate, altitude, and soil properties, *Cryptomeria* populations cultivated in varying geographical regions ranging from native Asian habitats to introduced populations in the Himalayas and the Azores Archipelago exhibit widely varying EO yields and compositions [6,55]. Comparative studies highlight significant fluctuations in the relative abundance of primary terpenes, such as shifting ratios of ⍺-pinene to cedrol, based on geographic origin [6]. These spatial variations underscore the strong gene-environment interactions that modulate the tree’s secondary metabolism, highlighting the phenotypic plasticity of the species in response to local ecological pressures [72].

Finally, the production of secondary metabolites is heavily dictated by tree age and the specific developmental stages of its organs [51,58]. Ontogenetic changes introduce significant temporal variability into the plant’s phytochemical profile. For example, the EO yield and chemical composition of female seed cones shift dramatically as they progress from immature to mature developmental phases, reflecting dynamic changes in reproductive metabolic demands [58]. Similarly, the concentration and complexity of volatile oils and non-volatile extractives accumulated within the solid wood evolve significantly as the tree ages. Mature trees frequently present a much more concentrated profile of bioactive volatile oils and defensive diterpenes compared to juvenile wood, optimizing the tree’s chemical defenses throughout its lifespan [51].

## 4. Uses: Pharmacological and Biological Applications

### 4.1. Traditional Medicine

Historically, the Chinese cedar has held significant cultural and ethnobotanical importance across East Asia. Before the advent of modern pharmacology, various parts of the tree, including its needles, bark, and heartwood, were extensively utilized in regional traditional medicine systems. Local populations recognized the therapeutic potential of the tree’s highly aromatic properties, frequently harvesting the foliage and wood to create natural remedies. These traditional formulations were primarily employed to manage common ailments, serving as an early testament to the plant’s inherent biological activity and beneficial secondary metabolites [50,73].

In traditional folk remedies, extracts and decoctions derived from Chinese cedar were frequently used to treat inflammatory conditions, respiratory issues, and skin disorders (Table 4). The resin-rich bark and leaves were typically steeped to produce infusions applied topically to wounds or fungal infections. This practice was intended to prevent infection and promote healing, leveraging the plant’s natural antimicrobial properties. Furthermore, the aromatic wood was utilized in living spaces not only for its structural durability but also for its perceived health benefits. Inhaling the fragrant volatile compounds released by the wood was historically believed to soothe the respiratory tract, reduce anxiety, and alleviate physiological stress [53,73].

Today, these historical ethnobotanical applications serve as a foundational guide for contemporary pharmacological research. Modern scientific investigations have begun to validate the traditional uses of *C. japonica* var. *sinensis* by identifying a rich matrix of phytochemicals, particularly diverse terpenoids and flavonoids, responsible for these health benefits. The transition from traditional folklore to evidence-based science has illuminated how ancient practices of utilizing Chinese cedar extracts successfully leveraged the plant’s complex biochemistry, paving the way for its current integration into modern biomedical and agrochemical fields [48]. A summary of these historical practices is provided in Table 4.

### 4.2. Antimicrobial and Antifungal Activity

Modern phytochemical screening has firmly established the potent antimicrobial properties of *C. japonica* var. *sinensis*, particularly against various bacterial pathogens. EOs and extracts derived from the foliage and twigs are rich in bioactive terpenoids that exhibit significant antibacterial efficacy against both Gram-positive and Gram-negative bacteria. Recent pharmacological evaluations have isolated multiple previously undescribed diterpenoids and sesquiterpenoids that demonstrate modest to strong inhibitory activity against several bacterial strains, including drug-resistant variants of *Staphylococcus* [48,59]. The broad-spectrum antibacterial nature of these botanical extracts highlights their potential as natural alternatives to synthetic antibiotics in combating infectious diseases.

In addition to antibacterial action, the extracts and volatile oils of the Chinese cedar possess strong antifungal activity. Volatile oils extracted from solid wood boards retain a high concentration of natural compounds that show marked in vitro efficacy against diverse human and plant pathogenic fungi [51]. Similarly, the EOs fractionated during the hydrodistillation of fresh foliage demonstrate targeted antifungal activity, effectively suppressing the proliferation of common phytopathogenic fungi as well as food-decaying microorganisms [51,59]. This robust antifungal profile provides a scientific basis for the tree’s historical use in preventing decay and treating topical fungal infections.

The antifungal defense mechanisms of *C. japonica* var. *sinensis* are particularly pronounced against wood-decaying fungi, which contributes to the exceptional natural durability of its timber. Specific abietane and pimarane diterpenoids isolated from the tree’s bark have been identified as primary active agents that inhibit the growth of wood-decay fungi [52,54]. By leveraging these intrinsic antifungal properties, researchers are actively exploring the use of these isolated compounds to develop eco-friendly treatments designed to protect commercially valuable agricultural crops and timber products from destructive fungal pathogens. Commercial EOs and hydrolates from forestry waste also demonstrate practical applications in preventing food deterioration caused by microbial growth [74].

### 4.3. Health and Biomedical Properties

The biomedical profile of Chinese cedar is characterized by exceptional antioxidant and anti-inflammatory activities. These health benefits are largely driven by its specific phenolic constituents. For instance, flavonoids identified in the needles, such as catechin and epicatechin, act as potent radical scavengers that protect cellular tissues from oxidative damage. Quercetin, another prominent flavonoid found in the species, is well-documented for its strong capacity to neutralize reactive oxygen species and downregulate inflammatory pathways. EOs obtained from sustainable forestry byproducts, such as sawdust and resin-rich bark, are highly effective at scavenging free radicals and mitigating oxidative stress through multiple biochemical pathways, as demonstrated by laboratory evaluations [75]. These robust antioxidant capacities are complemented by significant anti-inflammatory properties, where specific volatile compounds have been shown to suppress pro-inflammatory mediators in vitro and in silico [51,76]. Together, these activities underscore the potential of *C. japonica* var. *sinensis* extracts in managing chronic inflammatory conditions and preventing cellular damage associated with oxidative stress.

Emerging research has also illuminated the potent neuroprotective and anti-cholinesterase activities of the tree’s EOs, positioning them as promising candidates for neurodegenerative disease therapies. The volatile oils from solid wood and fresh foliage exhibit marked inhibitory effects against acetylcholinesterase and butyrylcholinesterase, which are key enzymatic targets in the management of Alzheimer’s disease [51,76]. Furthermore, inhalation of Japanese cedar wood EOs has been shown in clinical settings to increase salivary dehydroepiandrosterone sulfate (DHEA-S) levels. This physiological response is closely linked to inducing psychological relaxation and alleviating stress after monotonous work, highlighting the plant’s aromatherapeutic value [53].

Beyond neuroprotection, isolated natural products from the Chinese cedar exhibit a diverse array of additional biomedical applications. Various monoterpenes and diterpenoids have shown selective apoptotic and cytotoxic potential against certain cancer cell lines, presenting opportunities for novel chemopreventive drug development [77]. Additionally, the plant’s extracts contain specialized compounds demonstrating prominent anti-lipase activity, which could be beneficial for metabolic regulation [73]. This multifaceted pharmacological profile emphasizes the immense value of Chinese cedar in modern drug discovery and therapeutic development, driven by its unique chemical diversity.

### 4.4. Agrochemical and Biopesticide Uses

The agricultural sector is increasingly turning to *C. japonica* var. *sinensis* as a sustainable source of eco-friendly biopesticides and insect repellents. EOs derived from the tree have demonstrated high efficacy in repelling agriculturally significant arthropods and exhibit potent activity against disease-vectoring pests. Importantly, while these extracts are toxic to target pests, they can exhibit a high degree of selective safety for beneficial organisms. For instance, controlled, low concentrations of the leaf EO have been shown to provide immunostimulatory benefits to non-target pollinators, such as honey bees (*Apis mellifera*), modulating gene expression to enhance their resilience without causing toxicity [78].

Chinese cedar extracts are also highly regarded for their natural anti-termite properties, offering a viable alternative to toxic synthetic wood preservatives. Formulations containing the leaf extracts and wood-derived oils possess strong termiticidal activity, effectively deterring structural pests and preventing infestations [50,52]. The intrinsic chemical composition of the wood, which includes resilient secondary metabolites and specialized EOs, acts as a natural defense mechanism against both termites and fungal degradation. Consequently, botanicals derived from this species are being actively developed to protect commercial timber and agricultural infrastructure in an environmentally sustainable manner.

**Figure 4:**
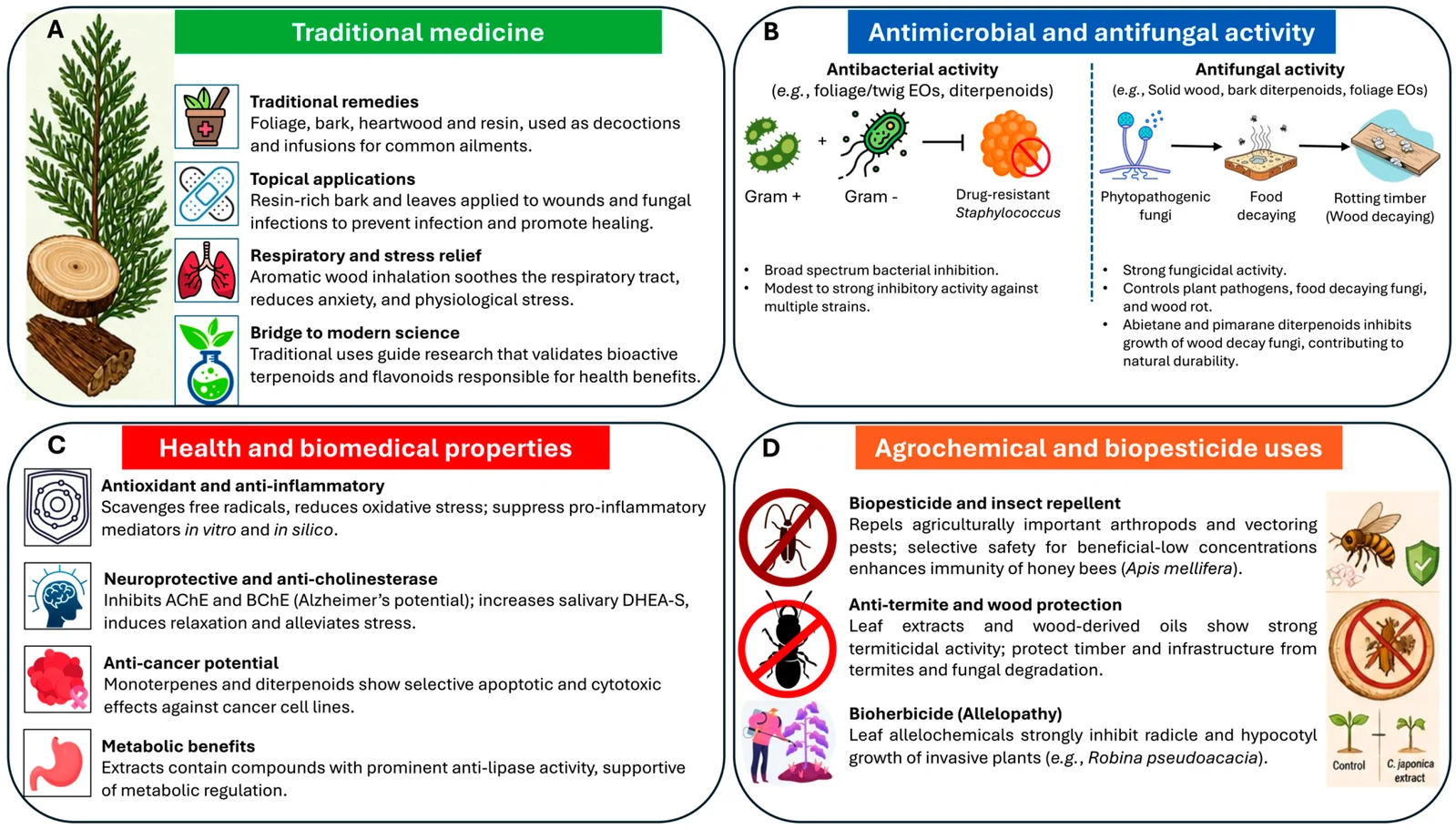
Application and bioactivities of *Cryptomeria japonica* var. *sinensis*. (**A**) Traditional medicine; (**B**) Antimicrobial and antifungal activity; (**C**) Health and biomedical properties; (**D**) Agrochemical and biopesticide uses. Key bioactive compound classes: Monoterpenes, sesquiterpenes; diterpenoids (abietane, pimarane); flavonoids; phenolics etc. AChE: acetylcholinesterase; BChE: butyrylcholinesterase; DHEA-S: dehydroepiandrosterone sulfate.

Furthermore, the broader agrochemical potential of *C. japonica* var. *sinensis* extends to targeted weed and pest management. Allelopathic compounds extracted from the leaves demonstrate significant growth-inhibitory effects on competing plant species. For example, leaf components strongly inhibit the radicle and hypocotyl growth of the invasive *Robinia pseudoacacia*, presenting opportunities for the development of natural bioherbicides [79]. By utilizing the biomass residues generated by the timber industry, such as fresh foliage and sawdust, the production of these diverse biopesticides represents a circular and highly sustainable approach to modern agriculture [59,79]. An overview of the diverse pharmacological, biomedical, and agrochemical bioactivities of Chinese cedar is summarized in Table 5 and Figure 4.

## 5. Uses: Forestry, Wood Properties, and Industry

### 5.1. Silviculture and Forest Management

The successful establishment and cultivation of Chinese cedar plantations rely heavily on optimized early-stage silvicultural practices. Proper soil conditioning and targeted fertilization during the seedling stage are crucial for accelerating initial growth, improving nutrient uptake, and ensuring high outplanting survival rates in afforested areas [80]. As seedlings mature into closed-canopy stands, initial planting density becomes a key determinant of long-term basal area growth and structural development [81]. To accurately monitor these growth dynamics, researchers have developed generalized nonlinear height-diameter models specific to Chinese cedar. These models equip forest managers with essential mathematical tools to predict biomass accumulation, optimize rotational planning, and evaluate overall plantation health [82].

Once the forest canopy closes, thinning emerges as a critical intervention for maintaining stand productivity and ecological balance. Adjusting thinning intensities, typically by removing 20% to 40% of the stand volume, drastically mitigates spatial competition, promoting rapid radial growth and diameter differentiation among the retained crop trees [83]. Beyond maximizing timber yield, moderate thinning significantly alters the microclimate of the plantation floor. The increased light penetration stimulates the diversity of understory vegetation, improves soil physicochemical properties, and revitalizes soil microbial communities, ultimately enhancing the long-term carbon storage capacity and resilience of the ecosystem [24].

Sustainable harvest management and canopy gap creation further ensure the long-term ecological stability of Chinese cedar stands. The strategic creation of forest gaps through selective logging directly influences localized soil microclimates, which boosts species diversity and facilitates natural regeneration within the plantation [84]. When clearcutting is necessary for timber extraction, modern silviculture advocates for strip clearcutting methods rather than massive block removals. Research indicates that optimizing the width of clearcut strips helps maintain the composition and stability of soil water-stable aggregates, thereby preventing structural degradation and soil erosion during subsequent planting rotations [85].

### 5.2. Wood Anatomy and Mechanics

The superior structural qualities and commercial value of Chinese cedar timber are fundamentally defined by its microscopic cellular anatomy and the genetic regulation of its wood formation. Micro-coring studies tracking intra-annual wood formation in humid subtropical climates reveal that the vascular cambium is highly responsive to seasonal temperature and moisture shifts, dynamically altering tracheid dimensions to produce distinct earlywood and latewood zones [22]. At the molecular level, secondary cell wall thickening and lignification are heavily governed by specific genetic networks. Transcriptomic profiling has identified critical candidate genes, such as *Cf4CL* and *CfCCoAOMT*, that dictate phenylpropanoid metabolism and lignin biosynthesis [86]. Additionally, transcription factors from the R2R3-MYB and NAC families act as master regulators of xylem development, providing vital targets for biotechnological breeding programs aimed at improving wood density [4,87].

Wood density is one of the most reliable predictors of the timber’s physical strength, stiffness, and mechanical resilience. Because traditional sampling is destructive, modern forestry increasingly utilizes the micro-drilling resistance method to evaluate standing trees. This non-destructive technology accurately estimates internal wood density, allowing foresters to assess the mechanical load-bearing capacity of living cedar trees prior to harvest [88]. Once harvested, the structural integrity of the timber is highly dependent on the intricate interaction between rigid lignin and flexible polysaccharides within the cell walls, which provide the essential compressive support required for structural applications [89].

Natural decay resistance and dimensional stability are equally vital for the longevity of the wood in commercial settings. While Chinese cedar exhibits a moderate degree of natural decay resistance against fungal degradation, this trait shows notable variation depending on the specific genetic clone and the environmental conditions of the stand [90]. Furthermore, the wood is sensitive to moisture absorption, which can cause swelling and mechanical weakening over time [91]. Comparative industrial studies demonstrate that treating the wood with superheated steam drying, as opposed to conventional kiln drying, drastically improves its dimensional stability and minimizes equilibrium moisture content, ensuring the mechanical integrity of the timber is preserved over its service life [92].

### 5.3. Industrial Timber Utilization

Renowned for its straight grain, excellent workability, and favorable strength-to-weight ratio, Chinese cedar is heavily utilized in commercial construction and the manufacturing of engineered wood products. To overcome the inherent softness and natural defects of solid softwood logs, the timber is increasingly processed into high-strength structural composites. Studies confirm the economic feasibility of utilizing domestic cedar for the mass production of laminated veneer lumber (LVL), which serves as a highly stable substitute for solid beams [93]. Furthermore, cross-laminated timber (CLT) panels manufactured from cedar layups exhibit robust out-of-plane bending performance and elasticity, making them ideal for load-bearing walls and floors in mid-to-high-rise sustainable architecture [94] (Table 6).

To further expand its utility in heavy-duty structural applications, the timber industry has developed advanced physical and chemical modification techniques. Thermo-mechanical hot pressing is used to densify the cellular structure of the wood, creating wood compression layered structural (WCLS) materials that boast vastly increased surface hardness, stiffness, and enhanced thermal conduction for energy-saving building elements [95]. Alternatively, “sandwich compression” methods allow manufacturers to selectively compress the exterior layers of the timber while maintaining a lightweight core, producing a highly dent-resistant material ideal for flooring and furniture [96]. For structural joints, impregnating the cellular matrix with acrylic or urethane resins significantly increases the wood’s partial compression and embedment properties, preventing cell wall collapse under heavy loads [108,109].

Beyond heavy construction, Chinese cedar is highly prized for indoor residential paneling, largely due to its unique sensory and physiological benefits. Solid wood boards of *Cryptomeria* naturally emit biogenic VOCs that possess documented antimicrobial, anti-inflammatory, and potential neuroprotective properties [51]. Exposure to these specific cedar-derived VOCs in indoor environments has been scientifically linked to positive psychophysiological effects. Controlled studies demonstrate that inhaling the aroma of cedar timber walls induces measurable physiological relaxation, lowers blood pressure, and reduces stress in human occupants, making it a premier material for wellness-focused interior design [110].

### 5.4. Bioenergy and Biomass

The extensive harvesting and milling of Chinese cedar generate massive quantities of lignocellulosic residues, including branches, sawdust, and bark, which serve as excellent feedstocks for the circular bioeconomy. Through advanced bioconversion techniques, the tough cellular matrix of cedar residues can be broken down into fermentable sugars for the production of sustainable liquid biofuels. Recent innovations utilizing simultaneous enzymatic saccharification and comminution (SESC) allow for the highly efficient extraction of intact lignin and lignin-polysaccharide complexes without the need for harsh, energy-intensive chemical pretreatments, preserving their natural chemical properties and structure for high-yield bioconversion [97]. Once extracted, the wood hydrolysates can be fermented by genetically engineered thermophilic bacteria, such as *Moorella thermoacetica*, to produce high yields of bioethanol [98].

Solid cedar residues are also heavily repurposed into advanced biocomposites and derived solid fuels. Mechanochemically acetylated cedar wood meal can be seamlessly blended with synthetic polymers to manufacture dimensionally stable wood-polypropylene composites. These lightweight, highly durable biocomposites are increasingly utilized as eco-friendly alternatives to pure plastics in automotive parts and outdoor decking [99]. Additionally, waste *Cryptomeria* wood can be pulverized and mixed with industrial byproducts, such as rigid polyurethane foams, to produce high-caloric mixed-waste briquettes. Analyses of the oxidation behavior and decomposition kinetics of these materials confirm their high combustion efficiency and suitability for industrial thermal power generation [111].

Finally, the industrial potential of Chinese cedar biomass extends into the extraction of high-value specialty biochemicals. During routine forest management, valuable EOs can be hydro-distilled from fresh foliage and twigs, yielding potent aromatic and antimicrobial compounds for the cosmetics and pharmaceutical industries [112]. Even residual lignin extracted via acid hydrolysis retains a wood-like cellular structure, allowing it to be recycled into novel structural biomaterials [113]. Intriguingly, specialized biotechnological processes have also been developed to ferment cedar wood sugars into flavorful, food-grade alcohols, capitalizing on the timber’s unique chemical profile to create novel wood-brewed liquors and commercial beverages [114].

## 6. Biotechnology, Genomics, and Molecular Breeding

### 6.1. Tissue Culture and Propagation

In vitro micropropagation serves as a critical foundation for the rapid multiplication of superior *Cryptomeria japonica* var. *sinensis* (Chinese cedar) genotypes. Traditional seed-based propagation in conifers is frequently constrained by long reproductive cycles and extensive genetic heterozygosity. To circumvent these limitations, tissue culture systems utilizing nodal segments, hypocotyls, and axillary buds have been meticulously developed to clone elite trees. The application of precise plant growth regulators is necessary to overcome the morphogenic recalcitrance that is often encountered during phase change and adult tree micropropagation [115]. These advanced micropropagation protocols secure a continuous, year-round supply of genetically uniform and high-quality planting stock for large-scale afforestation.

A pivotal component of practical mass clonal forestry is the successful adventitious rooting of vegetative stem cuttings. The underlying physiological and molecular mechanisms governing adventitious root formation in *Cryptomeria* have been increasingly elucidated through advanced transcriptomic profiling. For instance, microarray and RNA sequencing (RNA-seq) analyses have mapped distinct, temporal patterns of gene expression in the rooting zones compared to the aboveground parts of cuttings [116]. These specific gene clusters dynamically coordinate cellular dedifferentiation, hormone signaling, and cell wall biosynthesis to facilitate the emergence of root primordia. Such molecular insights are directly leveraged to optimize environmental conditions and exogenous hormonal treatments, thereby maximizing rooting efficiency in commercial nurseries.

Beyond whole-plant regeneration, specialized in vitro cell culture systems offer versatile platforms for fundamental biological research. Cell suspension cultures derived from young conifer needles have been successfully utilized as a stable in vitro model for inducing the formation of tracheary elements without requiring intact plants [117]. More recently, highly efficient ectopic xylem cell induction systems utilizing cultured somatic embryos have been established to unravel the complex molecular mechanisms underlying secondary cell wall formation [118]. These innovative in vitro systems not only deepen our understanding of conifer wood biology but also provide scalable cellular platforms essential for future genetic engineering applications.

### 6.2. Somatic Embryogenesis

Somatic embryogenesis (SE) represents one of the most vital areas of conifer biotechnology, offering a robust cellular platform for mass clonal propagation and genetic transformation. In *Cryptomeria*, the induction of embryogenic cell lines is typically initiated from immature zygotic embryos or seeds. Extensive studies have revealed that the successful initiation of SE and the subsequent proliferation of embryogenic tissues are strongly influenced by the specific genotype and inherent transcriptomic profiles of the explants [119]. Efficient SE initiation protocols have now been established across diverse genetic backgrounds, successfully utilizing explants derived from male-fertile, male-sterile, and polycross-pollinated seed families [100].

Following successful induction, embryogenic cell lines must undergo a rigorously controlled maturation phase to yield high-quality cotyledonary embryos. The efficiency of somatic embryo maturation depends on several critical culture variables, including the concentration of abscisic acid (ABA), the application of osmoticants like polyethylene glycol (PEG), appropriate amino acid supplementation, and specific basal salt formulations [101]. Optimizing these factors significantly maximizes the production of mature embryos that possess high germination capacities. Recent advancements have successfully combined these maturation protocols with marker-assisted selection (MAS) to efficiently mass-produce male-sterile somatic embryos, offering a biological solution to widespread cedar pollinosis [102].

To maximize the practical utility of SE in long-term tree improvement programs, ensuring the long-term preservation of elite embryogenic cultures is paramount. Prolonged subculturing often leads to an irreversible decline in embryogenic capacity and an increased risk of somaclonal variation. To mitigate this, reliable cryopreservation protocols have been developed, allowing elite embryogenic tissues of *Cryptomeria* to be safely preserved in liquid nitrogen for extended periods [103]. Upon thawing, these cryopreserved tissues rapidly resume active growth and successfully differentiate into somatic plantlets. This capability, combined with improved and simplified propagation systems, ensures that superior clonal germplasm can be securely archived and propagated indefinitely [120].

### 6.3. Omics Technologies

The application of high-throughput omics technologies has transformed the genomic understanding of *Cryptomeria*. Historically, the immense size and complex repetitiveness of conifer genomes posed significant barriers to molecular research. However, a major advancement was recently achieved by Japanese researchers with the assembly of a complete, chromosome-level reference genome for the model conifer *Cryptomeria japonica* (Japanese cedar), yielding an approximately 11 Gb genomic sequence [121]. At the organellar level, the complete chloroplast genomes of Chinese cedar (*Cryptomeria fortunei*, widely treated as synonymous with *C. japonica* var. *sinensis*) have been sequenced. Comparative analyses of these chloroplast genomes provide high-resolution insights into the phylogenetic relationships, population genetics, and geographic divergence of the species across East Asia [11].

RNA-seq has become an indispensable tool for deciphering the molecular responses of Chinese cedar to various abiotic and biotic stresses. For example, comprehensive transcriptome analyses of seedlings subjected to heat shock have successfully identified complex networks of heat-responsive genes that regulate survival under acute temperature extremes [122]. Similarly, integrated transcriptomic and metabolomic analyses conducted during cold acclimation have demonstrated that specific shifts in flavonoid and terpenoid biosynthesis pathways are directly responsible for winter needle discoloration and enhanced cold resistance [26]. These extensive stress-responsive transcriptomes serve as rich repositories for identifying candidate genes crucial for breeding climate-resilient cultivars.

Beyond stress adaptation, multi-omics approaches are increasingly employed to unravel the complex regulatory networks governing secondary growth and wood formation. RNA-seq profiling of the vascular cambium across different developmental stages has successfully isolated key candidate genes and transcription factors involved in secondary cell wall deposition and phenylpropanoid metabolism [4]. Concurrently, small RNA sequencing and degradome analyses of the cambial zone have identified specific microRNAs (miRNAs) and their target genes that dictate spatial and temporal cellular activity during wood development [123]. Together, these comprehensive omics datasets provide a precise molecular roadmap for understanding and improving high-quality timber traits in Chinese cedar.

### 6.4. Molecular Breeding and Genetic Engineering

Molecular breeding strategies have drastically accelerated the genetic improvement of Chinese cedar, effectively transitioning the field away from slow, traditional phenotypic evaluations. The deployment of high-throughput sequencing technologies, such as RAD-seq, has facilitated the discovery of abundant single nucleotide polymorphism (SNP) and simple sequence repeat (SSR) markers across the genome. A prominent application of these molecular markers is the precise MAS of pollen-free cedar varieties. Researchers have successfully fine-mapped male-sterility loci (e.g., *MS1*) and developed diagnostic markers, enabling the rapid, early-stage screening of hypoallergenic trees from large seedling populations [124,125].

Complementing MAS, the functional cloning and characterization of specific target genes have provided direct avenues for elite trait enhancement. Research on Chinese cedar has successfully isolated multiple functional genes associated with stress tolerance and wood quality. For example, the cloning and overexpression of the *CfICE1* transcription factor have been shown to dramatically amplify plant tolerance to cold, drought, and salt stresses in transgenic models [27]. Additionally, the identification of stay-green genes (*CfSGR1* and *CfSGR2*) helps regulate chlorophyll metabolism under severe abiotic stress [32], while the characterization of lignin biosynthesis genes (*Cf4CL* and *CfCCoAOMT*) offers promising functional targets for engineering customized wood density and structural properties [86] (Table 7).

The practical realization of advanced genetic engineering in *Cryptomeria* is supported by the establishment of robust cellular transformation platforms. Highly efficient *Agrobacterium*-mediated transformation protocols utilizing highly regenerative embryogenic tissue explants have been successfully established, providing a reliable bridge between tissue culture and genetic modification [104]. Leveraging this platform, scientists studying Japanese *C. japonica* have successfully implemented state-of-the-art CRISPR/Cas9 genome editing technologies. Recent advancements in the Japanese lineage include the targeted mutagenesis of the *CjACOS5* gene to genetically induce a completely pollen-free phenotype, alongside significant improvements in genome-editing efficiency achieved through Cas9 codon optimization [105,106,107]. While currently applied to Japanese populations, these biotechnological advances provide a precise genetic engineering roadmap that can be extrapolated to the Chinese lineage.

## 7. Conclusion and Future Perspectives

### 7.1. Summary of Findings

As a highly adaptable evergreen conifer of immense ecological and economic importance in southern China, the Chinese cedar is widely cultivated for its rapid growth, straight trunk, and excellent timber quality. These characteristics make it a foundational species for large-scale afforestation, landscaping, and terrestrial carbon storage. Beyond traditional forestry, recent studies have demonstrated its robust utility in environmental restoration, where it has been successfully employed to phytoremediate heavy metal-contaminated soils, augment soil organic carbon sequestration and rehabilitate microbial communities at degraded zinc smelting slag sites [126,127]. Furthermore, recent population genetic studies have revealed strong geographic variation and genetic differentiation within the species, underscoring its remarkable evolutionary capacity to adapt to diverse subtropical environments [3,40].

In addition to its ecological utility, Chinese cedar possesses a rich and valuable phytochemical profile. Integrated multi-omics analyses have successfully mapped the complex biosynthetic pathways of its secondary metabolites, particularly terpenoids and flavonoids. These studies have shown that the accumulation of these bioactive compounds is highly dynamic, fluctuating in response to seasonal changes and abiotic stresses. These specialized metabolites not only equip the tree with an intrinsic defense mechanism against environmental extremes and pests, but they also offer significant medicinal and aromatic value that can be extracted for commercial use [7,47].

Biotechnological research on this species has also made significant strides, providing a theoretical foundation for targeted genetic improvement. Transcriptomic profiling has identified numerous key functional genes, such as R2R3-MYB transcription factors that regulate secondary cell wall and lignin biosynthesis, and antioxidant enzymes like *CfICE1* that confer tolerance to cold, drought, and salt stress [34,87]. Concurrently, advances in tissue culture and SE have created reliable biological platforms for mass clonal propagation. Together, these breakthroughs bridge the gap between traditional selective breeding and high-efficiency, genomics-assisted improvement strategies.

### 7.2. Climate Change Impact

The accelerating pace of global climate change poses a significant threat to the natural distribution and future cultivation of *C. japonica* var. *sinensis*. Niche modeling predicts that shifting thermal regimes and increasingly erratic precipitation patterns will significantly restructure the suitable natural habitat for this species. As regional climates warm, the optimal growth zones for Chinese cedar are projected to shrink and migrate toward higher altitudes and latitudes, such as modeled shifts in the Dongting Lake watershed [17]. This environmental shift threatens to disrupt the local adaptation of current populations, potentially causing “genetic offsets”, characterized by an asynchrony between the extant genetic architecture and the shifting climatic envelope [128].

Erratic weather and rising temperatures are also expected to exacerbate biotic stresses. Warmer climates provide highly favorable conditions for the geographical expansion and prolonged activity of destructive forest pests. For example, simulations indicate an increasing overlap between the suitable habitats of Chinese cedar and severe defoliators like the *Dendrolimus houi* moth [129]. This synergistically amplifies the risk of devastating pest outbreaks, which could lead to severe germplasm loss and biomass reduction in both ancient natural forests and dense commercial plantations.

To secure the future of Chinese cedar cultivation, proactive afforestation strategies must be implemented to mitigate these climatic impacts. The species possesses inherent physiological plasticity such as the regulation of photoprotective pigments and stress-responsive genes under thermal extremes, but natural adaptation may be too slow [30]. Forest managers must urgently delineate new, climate-adjusted seed transfer zones to guard against the risks of genetic mismatch. By utilizing molecular markers to map climate-growth sensitivity, breeders can proactively select and deploy diverse, climate-resilient provenances, ensuring the long-term sustainability of afforestation efforts under shifting global temperatures [40].

### 7.3. Conservation Strategies

Despite the massive proliferation of Chinese cedar in commercial plantations, its ancient and wild genetic resources are facing a severe conservation crisis. Historical logging, habitat fragmentation, and heavy human interference have drastically reduced the footprint of the original, old-growth forests. Furthermore, widespread large-scale afforestation using a narrow genetic base of fast-growing clones has led to significant genetic introgression. This admixture dilutes the unique genetic signatures of native stands and threatens to permanently erase native adaptive traits that have evolved over millennia [2,40].

In situ conservation remains the most critical and urgent strategy for protecting the natural evolutionary processes of the species. It is vital to establish strict protections for relic natural populations, particularly ancient tree lineages located within protected areas like the Tianmushan Nature Reserve [2]. These ancient trees act as natural refugia, harboring rare genetic alleles that enabled the species to survive historical climatic shifts. Conservation policies must focus on preserving the integrity of these habitats by minimizing human disturbance and actively preventing the introduction of commercial pollen or non-native cultivars near these protected zones [3].

Complementing field protections, robust ex situ conservation programs must be drastically expanded to provide genetic insurance. The construction of comprehensive germplasm resource banks, seed banks, and living core collections is essential. By leveraging advanced high-throughput sequencing technologies like GBS and RAD-seq, researchers can accurately assess population structures and select core accessions that capture maximum genetic diversity with minimal redundancy [12,130]. Safeguarding these diverse genetic materials ensures that a rich pool of rare traits remains available for future scientific research, selective breeding, and ecological restoration.

### 7.4. Future Research Gaps

While substantial progress has been made in understanding the functional genomics and multi-omics of Chinese cedar, the application of advanced biotechnological tools remains a major research gap. Specifically, the implementation of precision genome-editing tools, such as the CRISPR/Cas9 system, remains in its infancy within conifer research. Because conifers have massive, highly complex genomes and are notoriously recalcitrant to standard *Agrobacterium*-mediated transformation, overcoming these biological bottlenecks is a critical future objective. Establishing highly efficient transformation and regeneration protocols will eventually allow for the rapid, targeted editing of genes controlling pest resistance, climate resilience, and wood quality, revolutionizing conifer breeding programs [1].

A second critical gap lies in the transition from fundamental phytochemical discovery to applied pharmacology. Current studies have extensively documented the rich secondary metabolites of Chinese cedar, noting the potent antioxidant, antimicrobial, and anti-inflammatory properties of its EOs and flavonoid extracts in in vitro settings [7,47]. However, as noted in the recent literature, comprehensive studies detailing the phenolic profiling (e.g., utilizing HPLC–MS) of this specific botanical variety remain surprisingly scarce. Characterizing the complete phenolic profile of this species represents a significant gap that must be addressed. Furthermore, there is a profound absence of rigorous in vivo toxicological evaluations and human clinical trials. Future research must bridge this translational gap by testing standardized botanical extracts in clinical settings to rigorously assess their safety profiles, pharmacokinetics, and therapeutic efficacy for commercial phytomedicine use.

Finally, deep comparative evolutionary and physiological studies between the Chinese variety (*C. japonica* var. *sinensis*) and the Japanese variety (*C. japonica* var. *japonica*) are highly needed. While both belong to the same relic species, thousands of years of geographical isolation and differing climatic pressures have resulted in distinct phenotypic traits, growth habits, and adaptive strategies [3]. Future research should prioritize comprehensive comparative genomics and reciprocal common garden experiments to resolve long-standing taxonomic debates and identify the specific genomic loci responsible for their respective environmental adaptations. Such knowledge will underpin collaborative, cross-varietal breeding strategies designed to leverage heterosis (hybrid vigor) [128].

## Figures and Tables

**Table 1:** Comparative morphological characteristics distinguishing the mainland Chinese lineage (*Cryptomeria japonica* var. *sinensis*) from the typical insular lineage (*C. japonica* var. *japonica*).

Morphological Feature	*Cryptomeria japonica* var. *sinensis* (Chinese Cedar)	*Cryptomeria japonica* var. *japonica* (Japanese Cedar)	References
Crown and branching habit	Diffuse habit; slender, flexible, and distinctly pendulous (drooping) branchlets.	More rigid and spreading branchlets.	[14]
Leaf (Needle) texture	Softer, longer, and thinner awl-shaped needles.	Rigid, stouter, and relatively shorter needles.	[14]
Branch insertion angle	Narrow spreading angle (typically 15° to 30°); strongly incurved toward the stem.	Wider spreading angle.	[10,14]
Male (Pollen) cones	Small, scaly; typically shorter than subtending leaves.	Generally equal to or longer than subtending leaves.	[14]
Female (Seed) cones	Possess fewer cone scales (rarely exceeding 20 scales per cone).	Typically bear 20 to 30 scales per cone.	[14]
Cone awns and bracts	Distal projections (awns) are shorter (1–2 mm); much smoother appearance.	Longer awns; distinctly spiny appearance.	[10,11]

**Table 2:** Phytochemical profiling of major non-volatile secondary metabolites in *Cryptomeria japonica* var. *sinensis*.

Phytochemical Class	Key/Representative Compounds	Primary Tissue Source	Major Biological/ Commercial Roles	References
Volatile monoterpenes	⍺-pinene, sabinene	Fresh needles (foliage)	Fresh pine aroma, broad-spectrum antibacterial action, biopesticidal properties.	[48,49,50]
Volatile sesquiterpenes	Cedrol, elemol	Leaves, sapwood, heartwood	Anti-inflammatory properties, anti-cholinesterase activity, fragrance industry value.	[48,49,50,51]
Abietane diterpenes	Sugiol, Ferruginol, 7-oxoabietane	Bark, heartwood	Potent antifungal activity against wood-decay fungi, antioxidant, and xanthine oxidase inhibition.	[52,53]
Pimarane diterpenes	Isopimaric acid, sclarene	Bark, sapwood	Anti-termite properties, notable enzyme-inhibitory capacities, cytotoxic potential.	[54,55]
Flavonoids (Aglycones and glycosides)	Catechin, Epicatechin, Quercetin, Rutin, Quercetin-3-*O*-rhamnoside	Needles (upregulated during winter)	Strong antioxidants; neutralize ROS; mitigate cold-induced oxidative damage.	[47,56]
Norlignans	Agatharesinol, Sequirin C	Heartwood	Imparts reddish-dark coloration to timber; enhances natural structural decay resistance against biological degradation.	[57]

**Table 3:** Chemical composition and profile of major volatile organic compounds isolated from *Cryptomeria japonica* var. *sinensis*.

Compound Class	Specific Compound	Primary Source Tissue	Olfactory Profile/Bioactivity	References
Monoterpenes	α-pinene	Fresh Needles, Twigs	Fresh, pine-like aroma; strong broad-spectrum antibacterial activity.	[48,49,50]
Monoterpenes	Sabinene, Limonene	Fresh Needles	Citrus/woody notes; contributes to biopesticidal and insect-repellent properties.	[49,59]
Sesquiterpenes	Cedrol	Heartwood, Foliage	Sweet, woody aroma; anti-inflammatory, sedative, and anti-cholinesterase properties.	[48,51]
Sesquiterpenes	Elemol	Sapwood, Leaves	Floral/woody notes; potent antifungal action against phytopathogens.	[50,58]
Sesquiterpenes	δ-Cadinene	Seed Cones, Wood	Herbaceous aroma; antioxidant and localized cellular defense.	[49,58]

**Table 4:** Traditional ethnobotanical uses of *Cryptomeria japonica* var. *sinensis* in regional folk medicine.

Plant Part Utilized	Traditional Preparation Method	Target Ailment/Traditional Use	Modern Pharmacological Correlation
Needles (Foliage)	Hot water decoction/Infusion	Drunk as a tea to treat respiratory issues, common colds, and mild internal inflammation.	High flavonoid content (antioxidant) and monoterpenes (antibacterial).
Bark/resin	Crushed into a topical paste	Applied directly to skin wounds, rashes, and localized topical fungal infections to prevent rot.	Abietane and pimarane diterpenes (strong antifungal/antimicrobial activity).
Heartwood	Burned as incense/Aromatic timber used in dwellings	Inhaled to soothe the respiratory tract, reduce anxiety, and alleviate stress and fatigue.	Volatile sesquiterpenes (e.g., cedrol) known to inhibit acetylcholinesterase and lower stress markers (DHEA-S).

**Table 5:** In vitro and in vivo pharmacological, biomedical, and agrochemical bioactivities of extracts and essential oils derived from *Cryptomeria japonica* var. *sinensis*.

Field of Application	Extract/ Compound Source	Target Organism or Mechanism	Observed Biological Effect	Lineage/ Source	References
Antibacterial	Foliage/twig EOs	Gram-positive and Gram-negative bacteria	Broad-spectrum bacterial inhibition, including efficacy against drug-resistant *Staphylococcus*.	Japanese lineage	[48,59]
Antifungal	Bark diterpenoids, solid wood and foliage EOs	Phytopathogenic, food-decaying, and wood-decaying fungi	Strong fungicidal activity; prevents agricultural diseases, wood rot, and food deterioration.	Japanese lineage	[51,52,53,59]
Neuroprotective and aromatherapy	Solid wood and fresh foliage EOs	Inhibits AChE and BChE; increases salivary DHEA-S	Enzyme inhibition (therapeutic potential for Alzheimer’s management); induces psychological relaxation and lowers stress.	Japanese lineage	[51,53,76]
Biopesticide and anti-termite	Leaf extracts, wood-derived oils	Structural pests (termites), arthropods, pollinators	Strong termiticidal activity; repels pests while acting as a selective immunostimulant for non-target honey bees (*Apis mellifera*).	Japanese lineage	[50,52,78]
Bioherbicide (Allelopathy)	Leaf allelopathic compounds	Radicle and hypocotyl growth	Strongly inhibits the germination and growth of invasive competing plants (e.g., *Robinia pseudoacacia*).	Japanese lineage	[79]
Anti-cancer and metabolic	Monoterpenes, diterpenoids	Cancer cell lines, metabolic lipases	Demonstrate selective apoptotic/cytotoxic potential and prominent anti-lipase activity.	Japanese lineage	[73,77]

**Table 6:** Advanced industrial utilization, biomass bioconversion, and biotechnological propagation platforms developed for *Cryptomeria japonica* and its Chinese variety (var. *sinensis*).

Sector	Technology/ Process Utilized	Output Product	Application/Key Benefit	Lineage/ Source	References
Structural architecture	CLT and LVL	Engineered wood panels and structural beams	Overcomes solid log softness; provides robust out-of-plane bending for load-bearing walls in mid-to-high-rise architecture.	Japanese lineage	[93,94]
Wood modification	Thermo-mechanical hot pressing	WCLS materials	Densifies cellular structure, vastly increases surface hardness, dent resistance, stiffness, and thermal conduction.	Japanese lineage	[95,96]
Bioenergy and biocomposites	SESC extraction and thermophilic bacterial fermentation	Bioethanol and wood-polypropylene composites	Converts tough lignocellulosic residues into fermentable liquid biofuels and dimensionally stable, lightweight bioplastics.	Japanese lineage	[97,98,99]
Clonal forestry	SE combined with cryopreservation	Cotyledonary somatic embryos	Yields a continuous, year-round supply of genetically uniform, elite, and male-sterile (pollen-free) planting stock.	Japanese lineage	[100,101,102,103]
Precision genetic engineering	*Agrobacterium*-mediated transformation and CRISPR/Cas9	Genetically modified, targeted mutant trees	Overcomes conifer transformation recalcitrance; creates precise gene knockouts for climate resilience and pollen-free traits.	Japanese lineage	[104,105,106,107]

**Table 7:** Summary of functionally characterized genes, key transcription factors, and diagnostic genetic markers isolated from *Cryptomeria japonica* (including var. *sinensis*).

Gene Symbol/ Locus	Gene Family/ Protein Type	Biological Role/Phenotypic Trait	Lineage/ Source	References
*CfICE1*	Transcription factor (Inducer of CBF expression 1)	Regulates cellular resistance to chilling injury; enhances cold, drought, and salt stress tolerance.	Chinese lineage	[27,33,34]
*CfAPX*	Cytosolic ascorbate peroxidase	Rapidly scavenges ROS; confers broad-spectrum tolerance to thermal and osmotic stresses.	Chinese lineage	[31]
*CfSGR1/CfSGR2*	Stay-green proteins	Mediates hormone signaling and regulates chlorophyll metabolism to prevent premature leaf senescence.	Chinese lineage	[32]
*CfCHLM*	Magnesium chelatase H subunit	Promotes essential chlorophyll synthesis; bolsters salt stress tolerance and physiological efficiency.	Chinese lineage	[33]
*Cf4CL/CfCCoAOMT*	Phenylpropanoid pathway enzymes	Dictates lignin biosynthesis and secondary cell wall thickening; prime targets for engineering wood density.	Chinese lineage	[86]
*MS1*	Male sterility locus 1	Fine-mapped diagnostic marker utilized for Marker-Assisted Selection (MAS) of hypoallergenic, pollen-free varieties.	Japanese lineage	[124,125]
*CjACOS5*	Acyl-CoA synthetase 5	Essential for pollen formation; targeted via CRISPR/Cas9 mutagenesis to induce a completely pollen-free phenotype.	Japanese lineage	[105,106,107]

## Data Availability

No new data were created or analyzed in this study. Data sharing is not applicable to this article.

## Abbreviations

ABA	Abscisic acid
Cas9	CRISPR-associated protein 9
Cf4CL	*Cryptomeria fortunei* 4-coumarate:CoA ligase
CfAPX	*Cryptomeria fortunei* cytosolic ascorbate peroxidase
CfCCoAOMT	*Cryptomeria fortunei* caffeoyl-CoA O-methyltransferase
CfCHLM	*Cryptomeria fortunei* magnesium chelatase H subunit
CfICE1	*Cryptomeria fortunei* inducer of CBF expression 1
CfSGR1/CfSGR2	*Cryptomeria fortune* stay-green 1/stay-green 2
CjACOS5	*Cryptomeria japonica* acyl-CoA synthetase 5
CLT	Cross-laminated timber
CRISPR	Clustered regularly interspaced short palindromic repeats
DHEA-S	Dehydroepiandrosterone sulfate
EO(s)	Essential oil(s)
GBS	Genotyping-by-sequencing
GC-MS	Gas chromatography-mass spectrometry
IR	Inverted repeats
LC-MS	Liquid chromatography-mass spectrometry
LVL	Laminated veneer lumber
MAS	Marker-assisted selection
miRNA(s)	MicroRNA(s)
MS1	Male sterility 1
NMR	Nuclear magnetic resonance
PEG	Polyethylene glycol
RAD-seq	Restriction site-associated DNA sequencing
RNA-seq	RNA sequencing
ROS	Reactive oxygen species
SE	Somatic embryogenesis
SESC	Simultaneous enzymatic saccharification and comminution
SNP(s)	Single nucleotide polymorphism(s)
SSR(s)	Simple sequence repeat(s)
VOC(s)	Volatile organic compound(s)
WCLS	Wood compression layered structural materials
